# Implementation of force distribution analysis for molecular dynamics simulations

**DOI:** 10.1186/1471-2105-12-101

**Published:** 2011-04-18

**Authors:** Wolfram Stacklies, Christian Seifert, Frauke Graeter

**Affiliations:** 1 CAS-MPG Partner Institute and Key Laboratory for Computational Biology, Shanghai Institutes for Biological Sciences, Chinese Academy of Sciences, 320 Yueyang Road, Shanghai 200031, China; 2 HITS gGmbH, Schloss-Wolfsbrunnenweg 35, 69118 Heidelberg, Germany

## Abstract

**Background:**

The way mechanical stress is distributed inside and propagated by proteins and other biopolymers largely defines their function. Yet, determining the network of interactions propagating internal strain remains a challenge for both, experiment and theory. Based on molecular dynamics simulations, we developed force distribution analysis (FDA), a method that allows visualizing strain propagation in macromolecules.

**Results:**

To be immediately applicable to a wide range of systems, FDA was implemented as an extension to Gromacs, a commonly used package for molecular simulations. The FDA code comes with an easy-to-use command line interface and can directly be applied to every system built using Gromacs. We provide an additional R-package providing functions for advanced statistical analysis and presentation of the FDA data.

**Conclusions:**

Using FDA, we were able to explain the origin of mechanical robustness in immunoglobulin domains and silk fibers. By elucidating propagation of internal strain upon ligand binding, we previously also successfully revealed the functionality of a stiff allosteric protein. FDA thus has the potential to be a valuable tool in the investigation and rational design of mechanical properties in proteins and nano-materials.

## Background

Cellular functions such as growth, motility, and signaling are often guided by mechanical forces [1-3]. How proteins distribute external mechanical stress largely defines their stability and function [4,5]. Being able to understand and predict the network of interactions defining the distribution of internal strain is a prerequisite for functional mutagenesis and rational design of function. A fundamental problem of most experimental and theoretical methods is their limitation to observing changes at the coordinate level. In many cases signals propagate without causing major atomic displacements, thereby hiding the communication pathway. We here present a new method termed force distribution analysis (FDA) that has the potential to overcome these limitations by observing changes in forces directly. Reminiscent of finite element analysis used to engineer macroscopic structures, FDA is capable to disclose the distribution of strain in molecular structures. FDA is entirely based on molecular dynamics (MD) simulations and can thus be carried out for any structure which can be subjected to MD. We have successfully demonstrated the application of FDA to proteins and biopolymers by revealing the mechanisms of force distribution, including the mechanically robust immunoglobulin domains [4], a stiff allosteric protein [5], the von Willebrand factor in blood vessels [6] and silk fibers [7].

**Figure 1:**
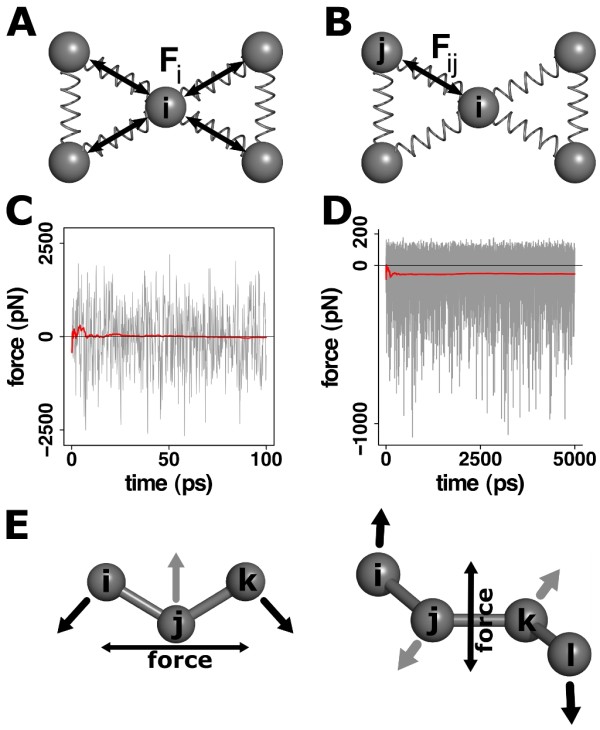
**The concept of pair-wise forces**. (A) Conventional MD uses the sum of all forces, , acting on a certain atom to derive the atomic motion. (B) In contrast, FDA works with pair-wise forces *F_ij_*, which is the force calculated between each pair of atoms *i*, *j *during an MD simulation. (C) The total force *F_i_*^atomic ^acting on an atom quickly decays to zero under equilibrium conditions. Observation of quickly decaying signals is not possible due to high equilibrium fluctuations. The plot shows only the *x *component of the *xyz *force vector for a single hydrogen bond O atom in the titin I27 domain [4]. (D) In contrast, even in equilibrium, pair-wise forces will not average to zero. This allows to compare different (equilibrated) states of a system. The plot shows pair-wise forces between the O-H atoms for the same hydrogen bond as in Figure 1 C. (E) Approximations used to transform multi-body forces into a pair-wise representation. Only the force acting along the direction of atoms *i*, *k *for angles and *i*, *l *for dihedrals is considered. This is sufficient to detect even minor re-arrangements.

FDA can be considered as a natural extension of any classical MD code. It allows to directly observe changes in atomic forces as a result of a perturbation. Examples for such perturbations are the application of an external force (pulling) or binding of a ligand. FDA makes use of pair-wise forces, i.e. the force an atom exerts on another atom. This is different from the total force acting on a certain atom, Figure 1A+B. By considering the direct force between each atom pair, the equilibrium force between these atoms can be different from zero, even for the theoretical case of a system without any motion. Atom-wise forces, i.e. the sum over all force vectors acting on an atom, instead average out to zero over time and are not of interest here. It is by observing pair-wise forces that we obtain the advantage to be able to detect signal propagation even through stiff materials where, by definition, forces propagate without causing major atomic displacement. A real world example is Newton's cradle. While coordinate changes and the corresponding ball-wise forces are non-zero only for the first and last ball, pair-wise forces are able to reveal the shock wave propagating through the stationary balls in-between as well.

During a typical MD run, forces that each two atoms exert on each other are calculated in every simulations step. These forces are directly summed up, resulting in a single force vector  acting on each atom. , averaging to zero in relatively short time, can not serve as a useful measure for the propagation of mechanical perturbations. FDA introduces a simple additional step to prevent this loss of information. Prior to the summation, pair-wise forces are stored in an *N *× *N *matrix, where *N *is the number of atoms.

## Implementation

The FDA code is implemented as an extension of Gromacs, version 4.0.5 [8]. Most of the functionality is kept in external libraries to ensure maximal modularity and portability. Gromacs uses so called "kernels", that is, highly optimized assembly loops, to calculate non-bonded interactions, which make up for most of the computation time. A slower fallback C/Fortran implementation is provided as well. Bonded interaction functions are implemented in C. Monitoring pair-wise forces required modification of both, the kernels and bonded interaction potentials. FDA-Gromacs thus currently only supports C kernels, though there is the option to use assembly loops where possible, e.g. for solvent-solvent interactions. On our test hardware, this resulted in a performance loss of up to 50%. Alternatively, however, FDA can be performed in a post-processing step on trajectories obtained with the standard MD code during re-run simulations, at only minor additional computational cost.

Translation and rotation forbid averaging of vectorized forces, and thus our implementation does not store force vectors, but rather the norm of those. Attractive and repulsive forces can still be distinguished by assigning opposite signs to them. In case vectorized forces are needed they can easily be recalculated from the trajectories. Most force fields make use of multi-body forces, typically angle and dihedral terms that involve more than two atoms. We use an approximation to transform angle and proper dihedrals into a pair-wise representation, see Figure 1E. Such a transformation is difficult for improper dihedrals, which for this reason are excluded. The same is true for long-range forces approximated with Particle Mesh Ewald (PME) [9], which cannot straightforwardly be transformed into pair-wise interactions and are thus excluded as well. At the typical cutoff distance of > 1 nm, the Coulomb potential already is relatively flat, i.e. slight changes in atomic distances have little effect on the force between them. Overall, we find most of the strain applied to a protein to be propagated via a network comprised of a series of short to medium ranged connections, i.e. the sub-nano scale [4,5].

Analogous to coordinate trajectories, pair-wise forces are stored in so called "force trajectories". FDA requires exhaustive sampling of the conformational space of the macromolecule to reduce noise (see below), and is thus currently based on time-averaged forces. The user may specify an output frequency; if this frequency is larger than the calculation step, forces will be averaged over this output interval. Force trajectories are stored in a binary file, containing a data block for each writing step. Each block contains the pair-wise forces, stored in a sparse matrix representation consisting of an index identifying the atom pair, the actual force and the interaction type. This way, it is possible to analyze contributions of every potential, typically bonded (bond, angle, dihedral) and non-bonded (electrostatic and van der Waals (VdW)) terms, separately. In order to separate Coulomb and VdW interactions, the upper matrix triangle (col > row) is used to store van der Waals forces, and the lower triangle (row > col) stores Coulomb forces. A detailed specification of the binary format is provided together with the FDA code.

Regarding setup and installation, there is no difference between FDA-Gromacs and the standard Gromacs distribution. Detailed installation instruction can be found on the offcial gromacs website, http://www.gromacs.org.

## Results and Discussion

One can use pair-wise forces to assess specific protein-ligand interactions, or to just debug a system, i.e. by checking for unrealistically high forces. Yet, in most cases, the response to a mechanical or allosteric signal becomes visible by comparing forces for different states. These states can be a system with and without applied external force, or the apo and holo configuration of an allosteric protein; here we call these states reference (ref) and perturbed (pert) for simplicity. Parts under mechanical strain become visible by subtracting forces of both states for each pair of interacting atoms. Changes in force, Δ*F*, are then given by the equation:(1)

To achieve a sufficient signal to noise ratio, FDA will often require exhaustive sampling of the conformational space. This is best done by calculating a set of independent trajectories, as MD simulations are prone to being trapped in local minima. The signal to noise ratio is estimated by comparing Δ*F *to differences in force observed for systems in the same state, Δ*F*^noise^, where Δ*F*^noise ^= *F*^ref ^- *F*^ref'^. Due to random coordinate fluctuations on the ps-ns timescale (Figure 1C), we found convergence of forces to require simulation timescales of 10 to several hundred nano seconds [4-7] (Figure 1D), depending on the flexibility of the system. Moreover, normalization by the standard error between independent trajectories, *ε*, may help to improve data quality. We previously defined the normalized change in force, Δ*f_ij _*as:(2)

### Visualization and statistical analysis

To be able to map changes in force onto the atoms of a protein structure, e.g. as a color gradient, a projection of the pair-wise space into the atom wise space is required. Such a projection is given by the simple column sum:(3)

**Figure 2:**
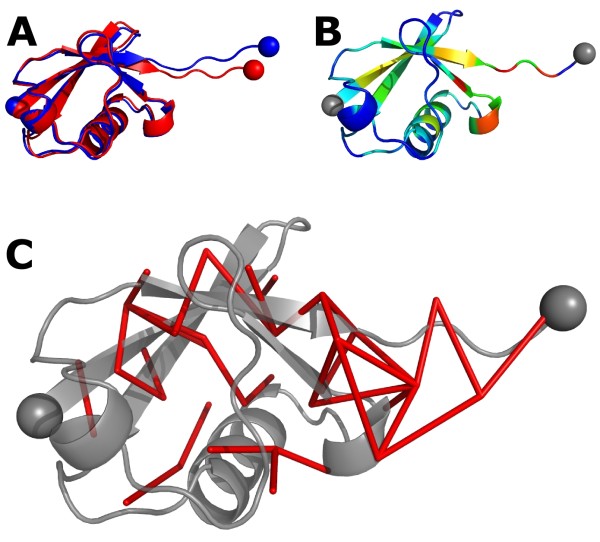
**Visualization of force distribution**. (A) A fit of ubiquitin (PDB code 1UBQ) with and without applied external strain. Only minor conformational re-arrangements are visible. (B) Color-coding of changes in pair-wise forces, Δ*F*, onto the protein structure reveals wide propagation of mechanical strain, even in parts showing no visible conformational change. Colors range from blue for Δ*F *= 0 to red for high Δ*F*. (C) Network representation of force distribution in ubiquitin. Edges represent residue pairs showing high change in pair-wise force.

Δ*F_j _*can be seen as the mechanical coupling of an atom *j *with respect to all other atoms. We use absolute values in the summation to avoid forces from canceling each other out. The Δ*F_j _*can be visualized by simply writing them as b-factors into a PDB file, Figure 2A+B. Alternatively, changes in pair-wise forces can be mapped as a network on a protein structure, Figure 2C. Pair-wise forces can be seen as edges connecting atoms, and the force between these atoms is the edge weight. The network of interactions under strain can then be visualized by drawing an edge between each atom for which |Δ*F_ij_*| > cutoff. Hereby it is often insightful to study the contributions of individual groups, such as side-chains or backbone atoms [4,6], what is easily achieved by restricting the summation to atoms within a certain group.

More details are obtained by statistical analysis on force trajectories, e.g. using principal component analysis (PCA). We previously identified a network of correlated fluctuations governing the allosteric function of MetJ by performing PCA on force trajectories [5]. In this case, it can be advantageous to look at forces between residue pairs instead of atom pairs. This will significantly decrease the memory requirements of the calculations.

Finally, FDA automatically provides detailed ligand-protein interaction profiles, as we have demonstrated for MetJ [5]. The interaction of a ligand with a protein structure depends on the complex interplay of individual atoms, charges play a crucial role. Intuitively, this can be seen as a pair-wise interaction between the ligand and the individual protein residues. FDA thus naturally provides a detailed description of the interaction pattern.

### Usage and tools

To ease the analysis of force trajectories, the FDA implementation comes with a tool called g_fdatools to process or convert force trajectories. g_fdatools provides options to sum up force trajectories, to average forces over a distinct time interval and to optionally calculate the corresponding variances. To allow for normalization and estimation of the sampling quality, the standard error *ε *between average forces obtained from individual runs can be calculated. Due to the vast number of pair-wise atomic interactions, which in the worst case grows quadratically with system size, it is often advantageous to instead analyze forces between residue pairs. g_fdatools allows to calculate residue averaged pair-wise forces  from atomic force trajectories in two ways.

The first and often sufficient way is to simply sum up pair-wise forces between every two residues *u*, *v *as defined in Equation 4.(4)

The resulting forces are not entirely correct, as we do not work with vectorized forces, but they are a good approximation. The second way is to calculate force vectors between residues using the coordinate trajectories. Here, prior to summation, exact atomic force vectors are restored by multiplying the force *F_ij _*with the coordinate vector  between atoms *i*, *j*:(5)(6)

In this case, x, y, z components and the norm of the force vectors between each residue pair will be returned. From our experience, a coordinate output frequency of 10 ps is sufficient to precisely restore the force vectors. In both cases, it is possible to restrict averaging to certain interaction types and atom groups, e.g. only Coulomb interactions between pairs of side-chains.

For further analysis we provide an R package called FDAtools that allows to import and analyze pair-wise forces in R [10]. The package provides several methods for data import, supporting full and sparse matrix representations. In all cases the output format is a simple matrix, which can easily be used for statistical analyses. Various functions to visualize the force distribution by mapping pair-wise forces onto PDB structures as described above are provided. Networks of residue pairs showing correlated changes in forces, as e.g. revealed by PCA, can easily be created with FDAtools as well. We hereto provide wrapper functions that allow to easily apply the R PCA implementation (prcomp) on FDA data. Finally, we provide a tool to convert binary force matrices into an ASCII representation, allowing users to easily import the data into the software package of their choice.

A step-by-step tutorial guiding the interested user through simulation setup, data-processing and analysis is available on the FDA project page hosted on Google Code http://code.google.com/p/force-distribution-analysis/.

### Limitations and complementary methods

FDA is perfectly fitted to elucidate mechanisms rendering proteins mechanically stable, or to understand slow conformational transitions. In cases of high intrinsic conformational flexibility or when larger conformational transitions occur during the sampling of forces, force averages will not converge, and FDA will fail to provide meaningful results. Such situations may occur for simulations at temperatures close to the melting point, for intrinsically disordered proteins, or molecules showing conformational transitions on the ps to ns time scale. Applying external strain to a protein and observing the induced changes by FDA as described in [4,6] is only feasible for mechanically stable and rigid proteins. For proteins with low mechanical stability, other mechanical perturbations such as e.g. ligand binding [5] can be characterized, or a time-resolved analysis of forces is required, which is the focus of ongoing work.

We here want to emphasize that FDA is not a replacement but a complement for existing, coordinate based methods. It provides an additional layer of information, namely how internal stress distribution will lead to conformational or entropic changes. By combining, for example, conventional trajectory analysis, normal mode analysis and FDA, we could shed light on dynamic allostery in MetJ, a transcription factor [5].

## Conclusions

We here presented a new tool, Force Distribution Analysis, as a way to track how perturbations distribute through molecules using standard Molecular Dynamics simulations. We outlined the basic assumptions, the implementation into the MD simulation package Gromacs, as well as the strength and limitations experienced in recent applications. Given the successful application of FDA to a number of proteins, we expect FDA to play a growing role in understanding and engineering the mechanics of other macromolecules and materials.

## Availability and requirements

Project name: force distribution analyis

Project home page: http://code.google.com/p/force-distribution-analysis/

Operating system(s): Linux/Unix

Programming language: C, additional tools in R

Other requirements: R (optional)

License: GNU GPL v2

Any restrictions to use by non-academics: none

## Authors' contributions

WS implemented all software and performed the analyses presented here. He also wrote this manuscript. CS is maintaining the project from now on, especially the R library. He also contributed to this manuscript. FG supervised the whole development process and contributed to this manuscript. All authors read and approved the final manuscript.
